# Public Awareness of Epilepsy and Social Attitude Toward Persons With Epilepsy Among the General Population in Makkah, Saudi Arabia

**DOI:** 10.7759/cureus.57398

**Published:** 2024-04-01

**Authors:** Rami Algahtani, Fayrouz Khateeb, Fawaz Khateb, Atheer S Alhazmi, Bashair A Alshareef, Bushra A Fatani

**Affiliations:** 1 Faculty of Medicine, Umm Al-Qura University, Makkah, SAU

**Keywords:** saudi arabia, practice, attitude, awareness, knowledge, population, epilepsy

## Abstract

Background: Epilepsy is a common neurological disorder characterized by an ongoing propensity to experience recurrent seizures. Public awareness varies significantly across different countries. The lack of awareness may lead to misconceptions, which in turn may affect the quality of care for these patients.

Aim: This study aims to assess public awareness and social attitudes toward patients with epilepsy among the general population in Makkah, Saudi Arabia.

Methods: A questionnaire-based cross-sectional study was conducted, targeting the general population of Makkah, Saudi Arabia. Data were collected anonymously from participants who met our inclusion criteria via electronic data collection Google Forms distributed through social media platforms. Questionnaires included participants' demographic data and details about their awareness regarding epilepsy, attitude, and anticipated behaviors. The eligible persons were asked to fill out the study questionnaire completely.

Results: A total of 1,126 eligible participants completed the study questionnaire. The participants' ages ranged from 18 to over 60, with a mean age of 32.1 ± 13.9 years. Of these participants, 849 (75.4%) were female. Interestingly, 97% of the participants reported that they had heard about epilepsy. A total of 96.7% do not think epilepsy is contiguous. Exactly 350 (31.1%) had an overall good knowledge and awareness regarding epilepsy. The most reported sources included relatives/friends (46.8%), internet (38.5%), and personal experience (27.9%). A total of 337 (29.9%) reported that they know how to deal with a seizure.

Conclusion: Our study revealed that the sampled population of Makkah is aware of epilepsy on a superficial level, but approximately one out each three participants in Makkah were knowledgeable regarding epilepsy and related causes overall. The overall attitude is positive. A well-targeted educational campaign, designed by physicians as trustful sources, is recommended.

## Introduction

Epilepsy is a neurological disorder that affects people all around the world [1]. It is characterized by recurrent seizures, which are short-lived episodes of involuntary movements that may include a part of the body or the entire body, occasionally accompanied by loss of consciousness and/or loss of control of bowel or bladder function [2]. Epilepsy is one of the most common neurological disorders affecting more than 50 million patients worldwide according to the World Health Organization [3]. In Saudi Arabia, the prevalence rate of epilepsy has been reported to be 6.54 per 1000 persons [4]. The social aspects of epilepsy may lead to impairment of adequate care and are a strong determinant of its clinical consequences. Due to stigmatization, patients and their families frequently choose not to seek treatment, even when it is available, because of the potential of associated discrimination [5-7]. Different religions, cultures, and ethnic groups have dissimilar levels of thought about epilepsy [8]. Numerous challenging ideas exist regarding epilepsy in many communities, and public awareness varies significantly between different cultures. As a result, these ideas about how epilepsy can bring suffering to patients and their families can result in a higher burden for epilepsy care [9-11].

We believe that, by providing the community with quality education and making more of an effort to increase public knowledge about getting appropriate treatment and seeking aid, we can improve patient care and quality of life and lower the rate of sudden unexpected death in epilepsy (SUDEP) in people with epilepsy [12,13]. In general, there is a lack of studies investigating the public awareness found within the western region of Saudi Arabia, leading us to conduct this assessment of the awareness, knowledge, and attitudes toward epilepsy in the western region of Saudi Arabia and the origins of more popular misconceptions.

## Materials and methods

Methodology

A descriptive cross-sectional study was conducted, targeting all participants in Makkah, Saudi Arabia. Raosoft (Raosoft, Inc., Seattle, WA) calculated the sample size to be 385 participants. Participants aged less than 18 years, healthcare professionals or academics related to any healthcare discipline, and health specialty students were excluded. We used an online pre-structured questionnaire to collect the data and classified patients according to their scores. The questionnaire was developed after intensive literature review and expert consultation in the study field. The questionnaire covered participants' personal data, family history of epilepsy, and history of exposure to epilepsy cases. The second section covered participants' knowledge and awareness regarding epilepsy and epileptic seizures. The third final section included participants' attitudes, perceptions, and anticipated behaviors regarding dealing with epileptic cases with proposed actions toward seizure cases. This study questionnaire was translated into Arabic and sent to the participants via social media platforms, namely, Whatsapp and X (previously Twitter), where their answers were recorded. The participants informed consent was obtained at the beginning of the questionnaire. A pilot study of 25 participants was conducted to assess the study questionnaire's validity and reliability with an estimated α-Cronbach's of 0.72. The study questionnaire was then made available to participants until no more responses were needed. Incomplete questionnaires were not included in the data analysis.

Data analysis

Data were collected, reviewed, and then imported into Statistical Product and Service Solutions (SPSS, version 21; IBM Corporation, Armonk, NY). All statistical methods were two-tailed with an alpha level of 0.05 and a significant P considered a P value of less than or equal to 0.05. Regarding public knowledge and awareness, each correct answer was given a one-point score. The overall knowledge level regarding epilepsy was assessed by summing up discrete scores for different correct knowledge items. If the total score was 60% or more of the total possible score, the level of knowledge was considered to be good, and scores less than 60% were considered poor. Descriptive analysis was done by evaluating frequency distribution and percentage for study variables, including participants' personal data, family history, and source of information. Participants' knowledge and awareness about epilepsy were also tabulated. The overall knowledge level was graphed. Participants' attitudes and anticipated behaviors towards epilepsy cases were then shown using frequency tables. Cross-tabulation for showing the distribution of the overall public knowledge and awareness in relation to personal data and other factors was evaluated using the Pearson chi-square test for significance and exact probability test if there were small frequency distributions.

## Results

A total of 1,126 eligible participants completed the study questionnaire. Participants ages ranged from 18 to more than 60 years, with a mean age of 32.1 ± 13.9 years old. Out of the total sample, 849 (75.4%) were females, and 1030 (91.5%) were Saudi. Regarding education, 737 (65.5%) were university graduates, and 287 (25.5%) had a secondary level of education or below. A monthly income of less than 5,000 SR was reported among 230 (20.4%) and 5,000-10,000 SR among 290 (25.8%), and 149 (13.2%) had a monthly income exceeding 20,000 SR. A total of 416 (36.9%) were unemployed, 326 (29%) were non-healthcare students, and 384 (34.1%) were healthcare employees. A total of 412 (36.6%) had at least one family member or friend suffering from epilepsy; 624 (55.4%) had seen a seizure before (Table 1).

**Table 1:** Socio-demographic data of study participants, Makkah region, Saudi Arabia. Total participants: 1,126

Socio-demographic data	No	%
Age in years		
18-20	207	18.4%
21-30	332	29.5%
31-40	193	17.1%
41-50	204	18.1%
51-60	131	11.6%
> 60	59	5.2%
Gender		
Male	277	24.6%
Female	849	75.4%
Nationality		
Saudi	1030	91.5%
Non-Saudi	96	8.5%
Educational level		
Secondary/below	287	25.5%
University graduate	737	65.5%
Post-graduate	102	9.1%
Monthly income		
< 5,000 SR	230	20.4%
5,000-10,000 SR	290	25.8%
10,000-15,000 SR	249	22.1%
15,000-20,000 SR	208	18.5%
> 20,000 SR	149	13.2%
Job title		
Not working	416	36.9%
Non-healthcare student	326	29.0%
Non-healthcare employee	384	34.1%
Is there any family member or friend who is suffering from epilepsy?		
Yes	412	36.6%
No	678	60.2%
Don’t know	36	3.2%
Have you seen a seizure before?		
Yes	624	55.4%
No	463	41.1%
Don’t know	39	3.5%

Table 2 shows that 97% (1,092) of study participants heard about epilepsy. A total of 96.7% (1,089) did not think that epilepsy is contiguous, 18% (203) disagreed that epilepsy affects sexual ability, and only 9% (101) reported that epilepsy did not affect pregnancy, while most participants do not know. As for age for those with epilepsy, 80.2% (902) know that it may affect all ages. Additionally, 62.9% (708) know that epilepsy is a brain disorder. Considering symptoms of epilepsy, 91.2% (1027) reported convulsions and muscle spasms, 42.1% (474) reported fainting, 38.5% (433) reported strange movements, and 15.5% (175) reported headaches. A total of 47.8% (538) of the study participants reported that epilepsy is not a dangerous disease, 63.7% (717) know about medications as a treatment for epilepsy, while 29.8% (335) do not know about treatment methods.

**Table 2:** Public awareness and knowledge regarding epilepsy, Makkah region, Saudi Arabia. Total responses of 1,126 participants.

Knowledge items		No	%
Did you ever hear about epilepsy before?	Yes	1092	97.0%
	No	34	3.0%
Do you think that epilepsy is contiguous?	Yes	12	1.1%
	No	1089	96.7%
	Don’t know	25	2.2%
Does epilepsy affect sexual ability?	Yes	283	25.1%
	No	203	18.0%
	Don’t know	640	56.8%
Does epilepsy affect pregnancy?	Yes	542	48.1%
	No	101	9.0%
	Don’t know	483	42.9%
Do you know a certain age for epilepsy?	Affect all ages	903	80.2%
	Children	18	1.6%
	Young	17	1.5%
	Elders	8	.7%
	I don’t know	180	16.0%
What is the cause of epilepsy?	I don’t know	260	23.1%
	Brain disease	708	62.9%
	Punishment from God	20	1.8%
	Evil eye or magic	139	12.3%
	Madness	37	3.3%
	Mental retardation	103	9.1%
	Genetic disease	406	36.1%
	Psychological disease	220	19.5%
	Without reason	101	9.0%
	Other	97	8.6%
Symptoms of epilepsy	I don’t know	39	3.5%
	Convulsions and muscle spasms	1027	91.2%
	Strange movements	433	38.5%
	Fainting	474	42.1%
	Headache	175	15.5%
	Hallucination	124	11.0%
Do you think epilepsy is more dangerous than?	Cancer	21	1.9%
	Diabetes	242	21.5%
	AIDS	21	1.9%
	Stroke	29	2.6%
	Neurological diseases	275	24.4%
	It is not dangerous	538	47.8%
Treatment of epilepsy	I don’t know	335	29.8%
	Medications	717	63.7%
	Surgery	112	9.9%
	Recitation of Quran	239	21.2%
	Herbal cauterization	64	5.7%
	Electric shock	91	8.1%
	Yoga and meditation	62	5.5%
	Others	71	6.3%

Regarding the level of public awareness and knowledge, a total of 350 (31.1%) had overall good knowledge and awareness regarding epilepsy, while 776 (68.9%) had suboptimal knowledge.

**Figure 1:**
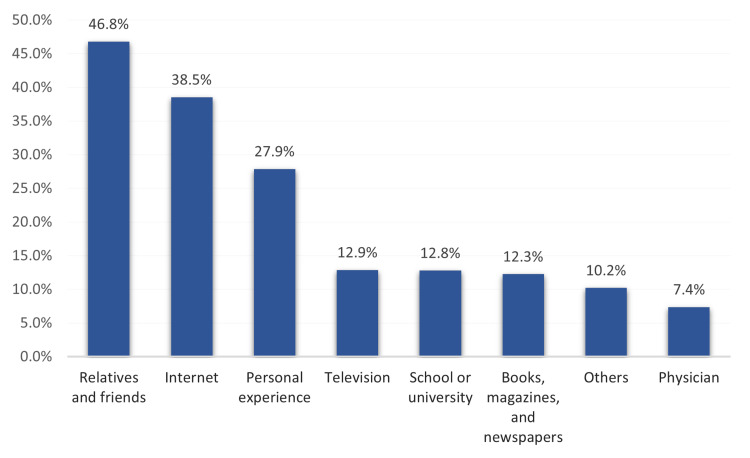
The main source of information regarding epilepsy among study participants.

The most reported sources of knowledge regarding epilepsy included relatives/friends (46.8%, 526), internet (38.5%, 433), personal experience (27.9%, 315), TV (12.9%, 146), school/university (12.8%, 145), and from physicians was the least reported source (7.4%, 83) (Figure 1).

A total of 89% (1,002) of the study participants stated that they would be a friend with a patient with epilepsy, a total of 84.4% (950) stated their willingness to go to a public place with an epilepsy patient, 84.1% (947) think that epilepsy patients can be an active member of society, and 23.1% (260) reported that they would be willing to personally marry and/or allow a son or a daughter to be married to someone with epilepsy. On the other hand, 63.1% (710) agreed that they would hire a patient with epilepsy if they were managers, and a total of 24.5% (276) were unsure, leaving 140 respondents (12.4% of 1,126 total) stating that, as a manager, they would not hire someone if they knew that individual had epilepsy. Finally, 26.4% (297) think that society treats epilepsy patients with stigmatization, and only 2.5% (28) think that epilepsy patients should be isolated from society (Table 3).

**Table 3:** Public attitudes towards epilepsy and epilepsy patients, Makkah, Saudi Arabia.

Attitude and perception	Agree		Disagree		Not sure	
	number	%	Number	%	number	%
Would you lie about epilepsy in a family member?	27	2.4%	1077	95.6%	22	2.0%
Do you think that epilepsy patients can be an active member of society?	947	84.1%	72	6.4%	107	9.5%
Do you think society treats epilepsy patients with stigmatization?	297	26.4%	592	52.6%	237	21.0%
Do you think that epilepsy patients should be isolated from society?	28	2.5%	1048	93.1%	50	4.4%
Is it possible to go to a public place with an epilepsy patient?	950	84.4%	86	7.6%	90	8.0%
If you were a manager, would you hire a patient with epilepsy?	710	63.1%	140	12.4%	276	24.5%
Would you marry or marry your son or daughter to a patient with epilepsy?	260	23.1%	474	42.1%	392	34.8%
Would you be a friend with a patient with epilepsy?	1002	89.0%	42	3.7%	82	7.3%
If you have a child, would you allow him to play with an epilepsy patient?	908	80.6%	110	9.8%	108	9.6%

A total of 337 (29.9%) reported that they know how to deal with a seizure. As for actions taken, the most reported correct actions included head protection (54.9%), directing the body and head to a side (40.1%), and staying near the person (40.1%) (Table 4).

**Table 4:** Participants' practice towards epilepsy patients during a seizure, Makkah, Saudi Arabia.

Practice	number	%
Do you know how to deal with a seizure if it occurs in front of you?		
Yes	337	29.9%
No	520	46.2%
Don’t know	269	23.9%
Actions for person who is having a seizure		
Head protection	618	54.9%
Fix the position of the person	466	41.4%
Direct the body and head to a side	452	40.1%
Stay near the person	393	34.9%
Tongue holding	366	32.5%
Spray water in the face	163	14.5%
Other	141	12.5%
Nothing	80	7.1%
Place a strong odor near the nose	68	6.0%
Shake the person	27	2.4%

The behaviors of study participants toward epilepsy patients varied. A total of 93.4% (1,052) think that epilepsy patient can continue their current or original education program. Again, however, a total of 260 (23.1%) stated that epilepsy patients "should study in a separate school." In addition, 90.9% (1,024) think that epilepsy patients can play sports, 89.5% (1008) think that epilepsy patients can get a job, 85.6% (964) think that epilepsy patients can marry, 83.1% (936) think that epilepsy patients can have children, 72.4% (815) think that the mental abilities of epilepsy patient are like others, and 60.4% (680) think that the lifespan of the epilepsy patient is like others (Table 5).

**Table 5:** Anticipated behaviors of study participants towards epilepsy patients.

Behaviors	Yes		No		Don’t know	
	Number	%	Number	%	Number	%
Do you think that the lifespan of the epilepsy patient is like others?	680	60.4%	109	9.7%	337	29.9%
Do you think that epilepsy patients can marry?	964	85.6%	43	3.8%	119	10.6%
Do you think that epilepsy patients can have children?	936	83.1%	43	3.8%	147	13.1%
Do you think that epilepsy patients can drive a car?	473	42.0%	397	35.3%	256	22.7%
Do you think that epilepsy patients can continue education?	1052	93.4%	33	2.9%	41	3.6%
Do you think that epilepsy patients should study in a separate school?	260	23.1%	731	64.9%	135	12.0%
Do you think that epilepsy patients can get a job?	1008	89.5%	41	3.6%	77	6.8%
Do you think that epilepsy patients can play sports?	1024	90.9%	32	2.8%	70	6.2%
Do you think that the mental abilities of epilepsy patients are like others?	815	72.4%	131	11.6%	180	16.0%
Do you think that epilepsy patient needs psychotherapy?	410	36.4%	395	35.1%	321	28.5%

Table 6 presents factors associated with public awareness and knowledge regarding epilepsy. A total of 38% (79) of participants with high income had an overall good awareness level compared to 27.8% (64) of others with low income with recorded statistical significance (P=0.044). Additionally, 37.1% (153) of those with a family member or friend who is suffering from epilepsy had a good awareness level compared to 28.6% (194) of others without (P=0.001). Good awareness was detected among 34% (212) of participants who witnessed seizures before in comparison to 28.9% (134) of others who did not (P=0.003). Similarly, 46.6% (157) of participants who knew how to deal with a seizure had a good knowledge level compared to 25.8% (134) of others (P=0.001).

**Table 6:** Factors associated with public awareness and knowledge regarding epilepsy. SR=Saudi Riyals. Significant p value <0.05 P: Pearson X2 test. $: Exact probability test. * P < 0.05 (significant)

Factors	Overall knowledge level				p-value
	Poor		Good		
	number	%	number	%	
Age in years					.190^$^
18-20	142	68.6%	65	31.4%	
21-30	217	65.4%	115	34.6%	
31-40	141	73.1%	52	26.9%	
41-50	136	66.7%	68	33.3%	
51-60	93	71.0%	38	29.0%	
> 60	47	79.7%	12	20.3%	
Gender					.362
Male	197	71.1%	80	28.9%	
Female	579	68.2%	270	31.8%	
Nationality					.156
Saudi	716	69.5%	314	30.5%	
Non-Saudi	60	62.5%	36	37.5%	
Educational level					.290
Secondary/below	208	72.5%	79	27.5%	
University graduate	497	67.4%	240	32.6%	
Post-graduate	71	69.6%	31	30.4%	
Monthly income					.044*
< 5,000 SR	166	72.2%	64	27.8%	
5,000-10,000 SR	214	73.8%	76	26.2%	
10,000-15,000 SR	165	66.3%	84	33.7%	
15,000-20,000 SR	129	62.0%	79	38.0%	
> 20,000 SR	102	68.5%	47	31.5%	
Job title					.064
Not working	290	69.7%	126	30.3%	
Non-health care student	209	64.1%	117	35.9%	
Non-health care employee	277	72.1%	107	27.9%	
Is there any family member or friend who is suffering from epilepsy?					.001*
Yes	259	62.9%	153	37.1%	
No	484	71.4%	194	28.6%	
3.00	33	91.7%	3	8.3%	
Have you seen a seizure before?					.003*^$^
Yes	412	66.0%	212	34.0%	
No	329	71.1%	134	28.9%	
Don’t know	35	89.7%	4	10.3%	
Do you know how to deal with a seizure if it occurs in front of you?					.001*
Yes	180	53.4%	157	46.6%	
No	386	74.2%	134	25.8%	
Don’t know	210	78.1%	59	21.9%	

## Discussion

Epilepsy is a common neurological disease [14]. According to the World Health Organization (WHO), “a diagnosis of epilepsy is reserved for those who have recurring seizures, at least two unprovoked ones” [15]. WHO estimates that eight people per 1,000 worldwide suffer from this disease [16], of which the prevalence in developed countries is less compared with developing countries. Despite substantial economic developments and improvements in health services have taken place, the prevalence of epilepsy is different between the countries, starting from 1.5 to 14.0 per 1,000 in Asia [17]. However, the results from Arab countries showed high prevalence. It was found that about 230 cases per 100,000 in Libya, and 650 per 100,000 in Saudi Arabia [4]. Regarding children, there are about 4-8 cases of 1000 children suffering from epilepsy in developing countries [18,19].

In Saudi Arabia, several studies have shown that there can be poor knowledge and negative attitudes toward epilepsy. Earlier studies reported that between 27% and 40% of participants attributed epilepsy to jinn possession [20,21]. A more recent study among people in the Aseer region revealed that 40.1% of the responders were convinced that it was the result of one or more spiritual reasons. More than 9% believed that treating epilepsy should be approached "spiritually". About 75% felt that epilepsy could be the result of a test delivered by God [22]. Another study in the Al-Qassim region showed that about all participants heard of epilepsy or convulsive seizures, 43.5% knew a person with the disease, and 48.4% had seen a person having a seizure. Additionally, two-thirds defined epilepsy as a brain disorder [23]. AlHarbi et al. conducted a systematic review in Saudi Arabia to assess public awareness of epilepsy and found that 6.4% of the population think epilepsy is a contagious disease; 90.7% have heard/read about epilepsy; 53.1% have witnessed a seizure; 38.6% think epilepsy is a psychological disorder; and 25.2% think it is caused by spirit possession [24]. A higher knowledge among teachers in Makkah was reported as 85.7% of responders knew that epilepsy is a neurological disease [25]. Internationally, Bain et al. [26] in Cameroon reported that most participants had heard or read about epilepsy, knew someone who had epilepsy, and had seen someone having a seizure. The most frequently cited cause of epilepsy was witchcraft. Most subjects believed epilepsy is contagious (in contrast to our study findings). Epilepsy was a form of madness or insanity for 33.5% of them. Kartal et al. [27] estimated that 68.4% heard or read about epilepsy, 44% knew someone with epilepsy, and 42.2% had witnessed a seizure. Additionally, Neni et al. [28] in Malaysia reported low public knowledge regarding epilepsy and its related causes. The situation is not much better in Hong Kong, where 58.2% had heard about epilepsy before. Of these, 55% had witnessed one or more epileptic seizures, and 18.9% knew one or more persons with epilepsy [29]. As for the source of information, the current study revealed that family and friends with the internet and personal experience were the most sources of information, whereas physicians were the least reported source, which means defects in performing their role. Higher knowledge was significantly reported among highly educated participants with higher income, those with a family history of epilepsy, and others who experienced a case with seizures and knew how to deal with it.

Considering participants' attitudes toward epilepsy cases, the overall trend is promising. The current study showed that most of them said that they would be a friend with a patient with epilepsy, think it is possible to go to a public place with an epilepsy patient, and think that an epilepsy patient can be an active member of society. Meanwhile, about one-fourth reported that they would marry or marry their son or daughter to a patient with epilepsy. On the other hand, about two-thirds agreed that they would hire a patient with epilepsy if they were managers, one-fourth think that society treats epilepsy patients with stigmatization, but only 2.5% think that epilepsy patients should be isolated from society. Similar findings were reported by Alsohibani et al. [23] as only 5.6% would protest their children associating with a person who occasionally experiences seizures, and a similarly small percentage (7.9%) would do so if their son or daughter decided to marry such a person. Similar findings integrating better attitudes toward epilepsy cases were also reported in the United Arab Emirates [30], Greece [31], and Taiwan [32]. A lower level of attitude was reported in China [33], Kuwait [34], and Jordan [35].

Our study has several limitations. The sampling was done in the Makkah region, which is one of 13 provinces that have considerable cultural variation, and hence generalization of our results over the Kingdom of Saudi Arabia may be limited. The study was conducted via an online questionnaire distributed through social media, which exposed the study to the risk of social-desirability bias and the potential impact of self-reporting data. In addition, social media dissemination of questionnaires cannot apply inclusion and exclusion criteria stringently. Alternative sampling for specific demographics, in-person survey, for example, may overcome this limitation. In addition, the methodology may result in under-reporting the results of men and people over the age of 50 given the online nature of sampling and questionnaire distribution.

## Conclusions

Our study revealed that the sampled population of Makkah is aware of epilepsy on a superficial level, but approximately one out of every three participants in Makkah were knowledgeable regarding epilepsy and its related causes overall. An in-depth analysis showed that better awareness was reported for symptoms and causes, while poor awareness regarding disease treatment was noticed. Higher knowledge was associated with having a family member/friend with epilepsy and experiencing a case with seizures. Physicians were the least reported source of information; hence, more effort should be channeled to them as trustful sources. The overall attitude toward epilepsy and its role in the community was positive but may need deeper analysis in regard to employment status. Acknowledging knowledge gaps is key to targeting trustful awareness techniques, which may vary based on the region of the Kingdom of Saudi Arabia as the population demographics and cultural beliefs are not uniform. An ongoing educational campaign, whether via social media or publicly in-person, is suggested to raise awareness and knowledge about epilepsy causes, symptoms, and complications and the available treatment options.
